# Call to action: the need for an incident learning system in Eastern Mediterranean Region and Turkey (EMRO+T)

**DOI:** 10.3332/ecancer.2025.2013

**Published:** 2025-10-14

**Authors:** Iqbal Alamri, Zahid Almandhari, M Saiful Huq

**Affiliations:** 1 Sultan Qaboos Comprehensive Cancer Care and Research Center, University Medical City, Muscat 123, Oman; 2 University of Pittsburgh School of Medicine and UPMC Hillman Cancer Center, Pittsburgh, PA, USA

**Keywords:** incident learning system, Eastern Mediterranean region and Turkey, patient safety

## Abstract

**Purpose:**

Modern radiotherapy is a complex, technology-driven process that requires precise planning and delivery to maximise tumour control and minimise harm to normal tissues. Errors in radiotherapy can result in underdosing tumours or overdosing healthy tissues, posing serious risks to patients. In the Eastern Mediterranean Region and Turkey (EMRO+T), variations in workforce expertise, institutional policies and regulatory frameworks contribute to inconsistencies in incident reporting and safety practices. While some centers adhere to international guidelines, others lack structured mechanisms for reporting and learning from errors. A regional incident learning system (ILS) could standardise reporting, enhance safety culture and improve patient outcomes.

**Methods:**

A cross-sectional survey assessed the state of radiotherapy ILS in EMRO+T. Ethical approval was obtained (CCCRC-94-2023), and the survey was distributed via Research Electronic Data Capture to 671 radiation oncology professionals. It gathered data on institutional classification, patient volume, technologies, local ILS presence, adherence to guidelines and perceptions of a regional ILS. Responses from 135 participants across healthcare settings were analysed.

**Results:**

Most respondents (66%) were from government hospitals, and 29.5% worked in private institutions. About 25% of centers treated over 2,000 patients annually, and advanced techniques such as intensity-modulated radiotherapy (75.2%) and stereotactic body radiotherapy (52%) were widely used. However, only 70.7% adhered to international reporting guidelines, and just 48.4% had a local ILS. Barriers included a lack of leadership support (51%) and confidentiality concerns. Although 62% supported reporting by all staff, opinions on confidentiality varied. Regular incident review occurred in 64% of institutions and 91.7% strongly supported a regional ILS.

**Conclusion:**

A regional ILS would foster knowledge-sharing, encourage a non-punitive culture and enhance transparency, accountability and continuous quality improvement in patient safety.

## Introduction

Modern radiotherapy is a highly sophisticated, technology-driven process that integrates advanced imaging, computer-controlled planning systems and artificial intelligence to deliver precise, high-dose radiation to malignant tumours while minimising exposure to normal tissues. The radiotherapy process consists of multiple steps, beginning with patient consultation and proceeding through multimodality imaging, treatment prescription, treatment planning, plan verification, treatment delivery and post-treatment follow-up, including palliative care. Patients receive radiation treatment using a variety of radiotherapy techniques, including three-dimensional conformal radiotherapy, intensity-modulated radiotherapy (IMRT), volumetric-modulated arc therapy, stereotactic radiosurgery and stereotactic body radiotherapy (SBRT) and brachytherapy. Each of these techniques involves distinct processes and technologies that influence how treatment is planned, verified and delivered. A multidisciplinary team of radiation oncologists, medical physicists and dosimetrists relies on cutting-edge imaging modalities such as computed tomography, magnetic resonance imaging and positron emission tomography to guide treatment planning. Using advanced treatment planning software, they optimise radiation dose distributions with millimeter or sub-millimeter accuracy, ensuring maximum tumour control while reducing the risk to healthy tissues.

The safe and accurate administration of radiotherapy is critical to achieving optimal clinical outcomes. Errors during any stage of the radiotherapy process—whether in imaging, contouring, treatment planning, verification or delivery—can lead to unintended consequences, such as underdosing tumours or overdosing normal tissues. Underdosing may compromise tumour control, while overdosing can lead to toxicity and long-term complications. Numerous studies have documented both minor and major radiotherapy errors, with varying degrees of patient harm, underscoring the need for robust quality assurance measures [1, 2]. The American Association of Physicists in Medicine (AAPM) Task Group 100 provides a comprehensive framework for assessing risks in radiotherapy processes, emphasising proactive risk management strategies to prevent errors at every stage of a radiotherapy process [3].

Given the complexity of modern radiation therapy, a proactive approach to error mitigation is essential. Incident learning systems (ILSs) provide a structured mechanism for identifying, analysing and learning from errors and near misses, strengthening safety culture and reducing preventable incidents. Many high-income countries have implemented national or institutional ILS frameworks tailored to their healthcare infrastructure. For instance, the International Atomic Energy Agency (IAEA) operates Safety in Radiation Oncology (SAFRON), a global voluntary reporting system for radiotherapy incidents [4]. The United Kingdom follows the National Reporting and Learning System, which collects data on patient safety incidents to inform best practices [5]. Similarly, the United States utilises the Radiation Oncology Incident Learning System, managed by the American Society for Radiation Oncology and the AAPM, to enhance safety and quality in radiotherapy [6]. However, the adoption of ILS programs in low- and middle-income regions remains inconsistent due to variability in resources, regulatory frameworks and institutional engagement.

The Eastern Mediterranean Region and Turkey (EMRO+T) exhibit substantial disparities in healthcare infrastructure, workforce expertise and access to advanced radiotherapy technologies. According to the IAEA’s Directory of Radiotherapy Centers, there are 448 radiotherapy centers in EMRO+T, equipped with 786 megavoltage machines and 117 brachytherapy services, though their capabilities vary widely [7]. While many individual centers may have their own incident reporting mechanisms and follow established safety procedures, these centers often operate in isolation, limiting opportunities for broader data sharing, benchmarking and region-wide learning. The establishment of a structured, regional ILS could facilitate collaboration among centers, enable data-driven policy changes and promote a consistent safety culture across diverse healthcare settings across the EMRO+T region.

This study aims to assess the need for a regional radiotherapy ILS in EMRO+T by conducting a survey across multiple radiotherapy centers. The survey evaluates existing safety practices, incident reporting mechanisms and institutional perspectives on the establishment of a structured learning system. By highlighting current challenges and gaps, this study advocates for the development of a comprehensive regional ILS to enhance patient safety and treatment quality across the region.

## Methods

We conducted a survey (IRB & EC Project ID: CCCRC-94-2023, Sultan Qaboos Cancer Care and Research Center, Muscat, Oman) to assess the current state of radiotherapy ILS in EMRO+T countries. The survey aimed to gather insights into existing safety practices, institutional capacities and perspectives on the need for a regional ILS.

The survey consisted of 23 questions, covering key aspects relevant to ILS implementation. The primary areas of inquiry included:

1. Classification of institutions (public or private hospitals, academic centers and specialised cancer institutes).
2. Number of patients treated annually to understand patient volume across different centers.
3. Available technologies, treatment techniques and modalities, including external beam radiotherapy and brachytherapy.
4. Existence of a local ILS, assessing whether institutions have structured incident reporting mechanisms.
5. Adoption of international standard guidelines for managing an existing local ILS.
6. Identification of personnel responsible for managing and reporting incidents within institutions.
7. Perceptions of the need for a regional ILS, evaluating support for a standardised system across EMRO+T countries.

### Survey data collection and management

Study data were collected and managed using Research Electronic Data Capture (REDCap), [8, 9] a secure, web-based software platform hosted at Sultan Qaboos Comprehensive Cancer Care and Research Center. REDCap is designed to support research data collection by providing:

1. An intuitive interface for validated data entry.
2. Audit trails to track data manipulation and export procedures.
3. Automated export functions for seamless integration with statistical software.
4. Interoperability with external data sources, facilitating standardised reporting. The use of REDCap ensured secure data handling and efficient management of survey responses.

### Survey distribution

The survey was distributed through targeted invitations to radiation oncologists, medical physicists and radiation protection officers across the EMRO+T region. It was disseminated via professional societies, including the Middle East Society of Radiation Oncology, the Middle East Federation of Organisations of Medical Physics and other regional organisations. These societies facilitated outreach to ensure broad participation from professionals directly involved in radiotherapy safety and incident reporting.

### Results

The survey instrument was distributed to 671 participants and 135 responses were received over a 2-month period. The majority of respondents (66%) were from government hospitals, demonstrating strong representation from the public sector, while 29.5% were from private hospitals. Nearly 25% of these institutions treat over 2,000 patients annually, reflecting a substantial healthcare burden.

Advanced radiotherapy techniques such as IMRT (75.2%) and SBRT (52%) were widely used, demonstrating a commitment to employing cutting-edge technologies for patient care. The survey also revealed that a significant number of facilities adhere to international guidelines for incident reporting, with 70.7% aligning with the IAEA’s safety standards. Additionally, 64.3% of institutions had well-established radiation safety programs and 77.2% had quality control programs in place.

**Figure 1.**
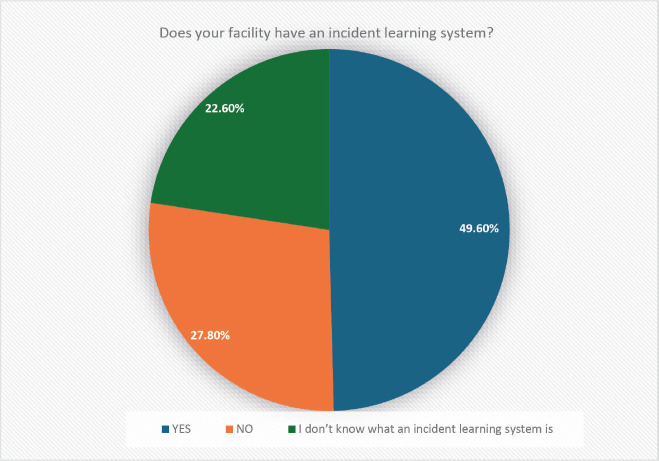
Percentage of responses to the question: ‘Does your facility have an ILS?’

However, the results also indicated gaps in ILS implementation. As shown in Figure 1, only 49.6% of institutions reported having an ILS in place, while 27.8% did not have an ILS and 22.6% of respondents were unaware of what an ILS is.

The lack of ILS adoption was attributed to several organisational challenges. The most commonly reported barriers were lack of leadership support (51.4%) and the absence of an institutional culture conducive to ILS implementation (45.7%).

Despite these challenges, 62% of respondents agreed that all staff members should report incidents. However, opinions on confidentiality varied:

- 47.5% preferred selective sharing of incident reports with designated staff members on a need-to-know basis.
- 41% supported full disclosure to all staff.
- 32% believed incident reports should be shared exclusively with senior management.

Despite these differences in reporting preferences, 64% of participants indicated that their institutions regularly review and analyse incident reports, highlighting an existing commitment to learning from errors.

**Figure 2.**
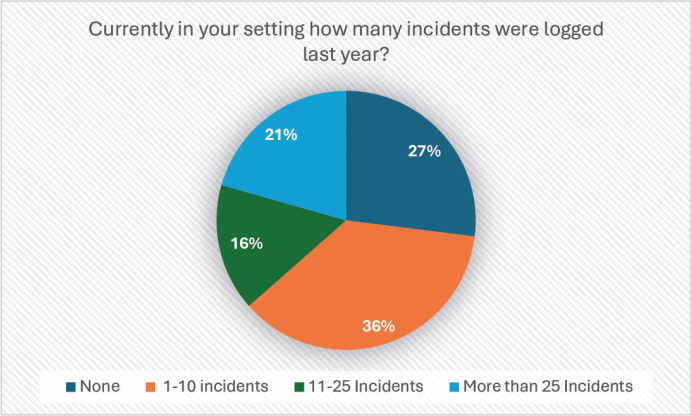
Percentage of responses to the question: ‘How many incidents were logged last year in your setting?’

The survey also assessed the number of incidents reported in the previous year (Figure 2). The data showed that incident reporting remains low, with 27% of respondents reporting zero incidents and only 36% indicating that between 1 and 10 incidents were logged during the review period. This suggests that the culture of incident reporting needs further encouragement, particularly through leadership-driven initiatives.

The most significant finding of this survey was the overwhelming support for the establishment of a regional ILS across EMRO+T countries. A remarkable 91.7% of respondents strongly agreed that a regional ILS should be developed. Following these findings, various regional societies have committed to taking a leadership role in developing an ILS for the region.

## Discussion

The survey results provide a comprehensive overview of radiotherapy practices, compliance with incident reporting and existing challenges in ILS implementation across EMRO+T countries. While many institutions follow international safety guidelines and utilise advanced technologies, barriers to ILS adoption persist.

Existing literature highlights that radiotherapy errors often stem from multiple factors, including poor decision-making, human errors, lack of peer-review processes, inadequate quality management programs and the absence of structured radiation safety protocols. An effective ILS is crucial for identifying errors, analysing their root causes and implementing corrective actions to enhance patient safety.

A well-functioning ILS should enable healthcare facilities to systematically identify, report and analyse errors, near misses and adverse events in healthcare, particularly in radiotherapy. Its primary goal is to enhance patient safety by shifting from a punitive approach to a learning-oriented culture, where errors are seen as opportunities for improvement rather than blame. By standardising processes and disseminating lessons learned, an ILS helps refine protocols, reduce errors and promote best practices across institutions. This learning-oriented approach fosters a culture of safety, continuous improvement and high-quality patient care.

Across EMRO+T, there is significant variability in clinical practices due to differences in healthcare infrastructure, expertise and training opportunities. While many centers have adopted advanced techniques like IMRT (75.2%) and SBRT (52%), these technologies introduce greater complexity in the entire treatment process. As a result, the potential for patient harm increases if major errors occur at any point along the treatment process. However, there is limited published data on the number and severity of incidents occurring in radiotherapy clinics across the region. The lack of structured reporting mechanisms and fear of blame or punitive actions may contribute to underreporting.

Furthermore, there is little to no culture of sharing lessons learned from incidents within the region. The survey findings support this observation, with only a minority of institutions actively logging incidents. Addressing this gap requires frequent audits, peer reviews, enhanced education and training programs and the establishment of robust quality management frameworks.

Despite these systemic barriers, the survey revealed strong support for creating a regional ILS, with 91% of respondents advocating for its establishment. This demonstrates a collective commitment to improving patient safety through cross-border collaboration and shared learning.

The implementation of a regional ILS will help shift the focus of incident reporting from a punitive approach to a learning-oriented, non-punitive system. This empowers healthcare professionals to report incidents without fear of retribution, fostering an environment of transparency, accountability and continuous improvement. By promoting knowledge sharing and best practices, a regional ILS would significantly enhance patient safety across EMRO+T clinics.

## Conclusions and call to action

A necessary outcome of this study is the urgent need to establish a regional ILS across the EMRO+T. Given the strong consensus among radiation oncology professionals, stakeholders – including healthcare institutions and professional societies – must take coordinated action to develop and implement a structured ILS framework.

This regional initiative will facilitate collaboration, promote standardised safety practices and ensure that lessons learned from incidents contribute to the continuous improvement of radiotherapy practices across the region.

As a next step, various regional societies should spearhead the development of a regional ILS, leveraging existing international frameworks such as SAFRON to create a tailored, region-specific system. Establishing this system will not only improve patient safety but also foster a stronger culture of learning and accountability in radiation oncology across EMRO+T.

## Conflicts of interest

There are no conflicts of interest.

## Funding

There was no funding.
